# Stress-induced chaperones: a first line of defense against the powerful oxidant hypochlorous acid

**DOI:** 10.12688/f1000research.19517.1

**Published:** 2019-09-23

**Authors:** Camille V. Goemans, Jean-François Collet

**Affiliations:** 1 European Molecular Biology Laboratory, Meyerhofstrasse 1, 69117, Heidelberg, Germany; 2 de Duve Institute, UCLouvain, Avenue Hippocrate 75, 1200 Brussels, Belgium

**Keywords:** oxidative stress, bleach, polyphosphate, bacteria, protein folding, chaperone, holdase, chlorination

## Abstract

Hypochlorous acid (HOCl; bleach) is a powerful weapon used by our immune system to eliminate invading bacteria. Yet the way HOCl actually kills bacteria and how they defend themselves from its oxidative action have only started to be uncovered. As this molecule induces both protein oxidation and aggregation, bacteria need concerted efforts of chaperones and antioxidants to maintain proteostasis during stress. Recent advances in the field identified several stress-activated chaperones, like Hsp33, RidA, and CnoX, which display unique structural features and play a central role in protecting the bacterial proteome during HOCl stress.

## Introduction

Like all aerobic organisms, bacteria naturally produce reactive oxygen species (ROS) as metabolic by-products, for instance during electron transfer in the respiratory chain. The addition of one electron to O _2_ leads to the production of the superoxide radical (O _2_^•–^), a toxic compound, which dismutates to form hydrogen peroxide (H _2_O _2_) and molecular oxygen (O _2_) either spontaneously or via catalysis by superoxide dismutases ^1– 3^. H _2_O _2_ can then react with ferrous iron to generate more reactive hydroxyl radicals ( ^•^OH) by the Fenton reaction. These oxidizing molecules can damage cellular components including DNA, membrane lipids, and proteins, which can lead to cell death. Therefore, bacteria have evolved defense mechanisms, which include enzymes, such as catalases and peroxiredoxins, that directly react with ROS to convert them to harmless products, and repair enzymes, such as thioredoxins and methionine sulfoxide reductases, that catalyze the reduction of oxidized amino acids in damaged proteins. For more information on the mechanisms that allow bacteria to cope with oxidants and rescue oxidatively damaged proteins, we refer the reader to a recent review in which we discuss the role of the thioredoxin and glutaredoxin systems and highlight the importance of protein repair in bacterial physiology and virulence ^4^.

Because of their toxicity, it is not surprising that the immune system of multicellular eukaryotes uses ROS as weapons to kill bacteria. When bacteria enter a tissue, the inflammatory response is turned on and phagocytes (neutrophils and macrophages) are recruited to the site of infection ^5^. These cells, whose cytoplasm is filled with lysosomal granules containing a variety of bactericidal and digestive enzymes ^6, 7^, are able to engulf bacteria. After phagocytosis, the phagosome and the granules fuse, forming a phagolysosome ^6, 7^. Then, high levels of ROS (O _2_^•–^ and H _2_O _2_) are produced in a phenomenon known as “oxidative burst” ^6– 8^, strongly contributing to the killing of the bacterium.

## Hypochlorous acid, an oxidative weapon to combat invading bacteria

In neutrophils, ROS production induces the release of myeloperoxidase (MPO), a glycoprotein stored in the phagocyte granules, into the phagolysosome. This enzyme converts H _2_O _2_ and chloride into hypochlorous acid (HOCl) ^5^, a strong oxidant (E ^0^’ [HOCl/Cl ^–^] = 1.28 V) that is also the active ingredient of household bleach, the most widely used disinfectant. HOCl is extremely effective and reacts with most macromolecules, including lipids, cholesterol, NADH, nucleotides, and proteins ^9– 11^. In contrast to H _2_O _2_, which can diffuse through membranes ^12^ and has a substantially longer lifetime (10 µs; ^13^), HOCl acts rapidly and locally, with a lifetime of ~0.1 µs ^14^ and a short diffusion length *in vivo* (0.03 µm when it reacts with cysteines and methionines ^15^). Thus, by catalyzing the conversion of long-lived, diffusible H _2_O _2_ into locally confined HOCl, MPO contributes to the prevention of collateral tissue damage during oxidative burst, allowing the specific targeting of the engulfed bacterial pathogen ^14^.

## Proteins are favorite HOCl targets

**Figure 1.**
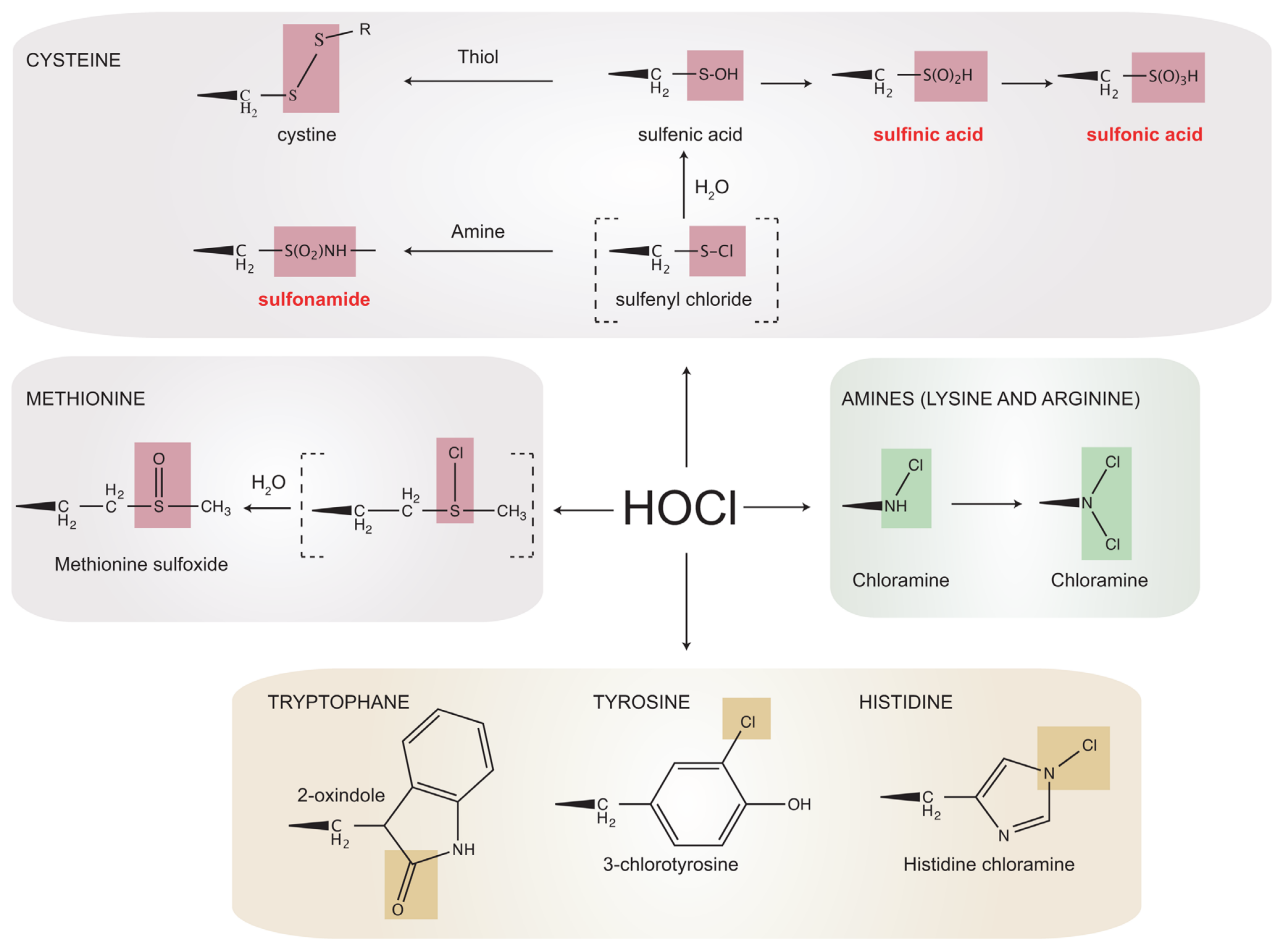
Side-chain modifications observed during hypochlorous acid (HOCl) stress. HOCl modifies the side-chains of several amino acids. Reaction with the thiol group of cysteine residues leads to the formation of an unstable sulfenyl chloride. Sulfenyl chloride quickly reacts with water to form a sulfenic acid or with primary and secondary amines to form sulfonamide crosslinks, which are irreversible. Sulfenic acids can be reduced back to a thiol by the cytoplasmic reducing systems, be further oxidized to sulfinic or sulfonic acids that are irreversible and lead to protein inactivation and degradation, or react with another thiol to form disulfide bonds. Irreversibly oxidized forms are indicated in red. HOCl also reacts with methionine residues to form methionine sulfoxides. Primary and secondary amines (lysine and arginine) are the second targets of HOCl in proteins, which chlorinates them to form chloramines (the secondary amine of arginine is not shown). The imidazole ring of histidine reacts with HOCl to form a short-lived chloramine, which rapidly transfers its chlorine group to another amine. Tryptophan reacts with HOCl to form 2-oxindole while reaction of HOCl with tyrosine forms 3-chlorotyrosine.

Although HOCl targets all cellular components, proteins, because of their reactivity and high abundance, are thought to be its primary target. The oxidation of amino acid side-chains in proteins ( Figure 1) can cause the loss of secondary or tertiary structure, thereby impacting protein stability and activity. HOCl reacts extremely quickly (k≈ 3 × 10 ^7^ M ^–1^. s ^–1^) with sulfur-containing residues (cysteines and methionines) ^10, 11, 16^. Cysteine thiols are first rapidly chlorinated to form a sulfenyl chloride, an unstable intermediate that can react with water to form a sulfenic acid (R-SOH) ( Figure 1). Most sulfenic acids are highly unstable (half-life in minutes ^17^) and either react with a cysteine thiol present in the vicinity to form a disulfide, whose formation is in principle reversible by the action of an oxidoreductase like thioredoxin ^18^, or are further oxidized to sulfinic (R-SO _2_H) and sulfonic (R-SO _3_H) acids ( Figure 1), two irreversible modifications that typically cause protein inactivation and degradation. Degrossoli and co-workers showed that exposure of bacteria to the oxidant mixture released during phagocytosis causes a rapid and massive oxidation of thiols ^19, 20^. By taking advantage of fluorescent redox-sensitive protein probes expressed by the engulfed bacteria, they highlighted the critical role of MPO-generated HOCl in the toxic oxidizing cocktail released by immune cells ^19^.

Methionines can be oxidized to methionine sulfoxides (Met-SO), and this oxidation is likely to play a critical role in the bactericidal action of HOCl, as strains lacking methionine sulfoxide reductases, enzymes that reduce methionine sulfoxides back to methionine, become more sensitive to HOCl ^21^. In line with this idea, we recently identified an enzymatic system expressed in the cell envelope of Gram-negative bacteria that participates in the defense mechanisms against HOCl by reducing oxidized methionine residues in this compartment ^22^. This system involves the molybdenum-containing enzyme MsrP and the heme-binding membrane protein MsrQ and uses electrons from the respiratory chain for methionine rescue. Remarkably, MsrP and MsrQ are specifically induced by HOCl in *Escherichia coli*, and not by H _2_O _2_, which further highlights the physiological need for cellular systems devoted to the defense against HOCl ^22^.

In addition to sulfur-containing residues, primary ( Figure 1) and secondary amines (not shown) are also susceptible to HOCl, which chlorinates them to form chloramines (k≈ 10 ^3^–10 ^5^ M ^–1^. s ^–1^) ^10, 11, 16^. Tryptophan is also thought to react with HOCl to form 2-oxindole, but how these molecules form remains unclear ( Figure 1) ^10, 11^. The imidazole ring of histidine reacts with HOCl to form a short-lived chloramine, which rapidly transfers its chlorine group to another amine. Finally, the chlorination of tyrosine into 3-chlorotyrosine is a marker used to detect HOCl-induced damage ( Figure 1) ^10, 11^.

## Stress-activated chaperones protect bacteria against HOCl-induced protein aggregation

The mechanism by which HOCl contributes to bacterial killing in the phagolysosome is not fully understood ^5^. However, it is thought to be a combination of events including oxidation-induced protein aggregation ^23^ and a drastic decrease in cellular ATP caused by the inactivation of the F _1_-ATP synthase, loss of glucose respiration, and the formation of polyphosphate (PolyP) ^24^. The bactericidal activity of HOCl can also be explained by the loss of activity of GroEL (Hsp60), an essential chaperone inactivated upon HOCl treatment ^25, 26^.

**Figure 2.**
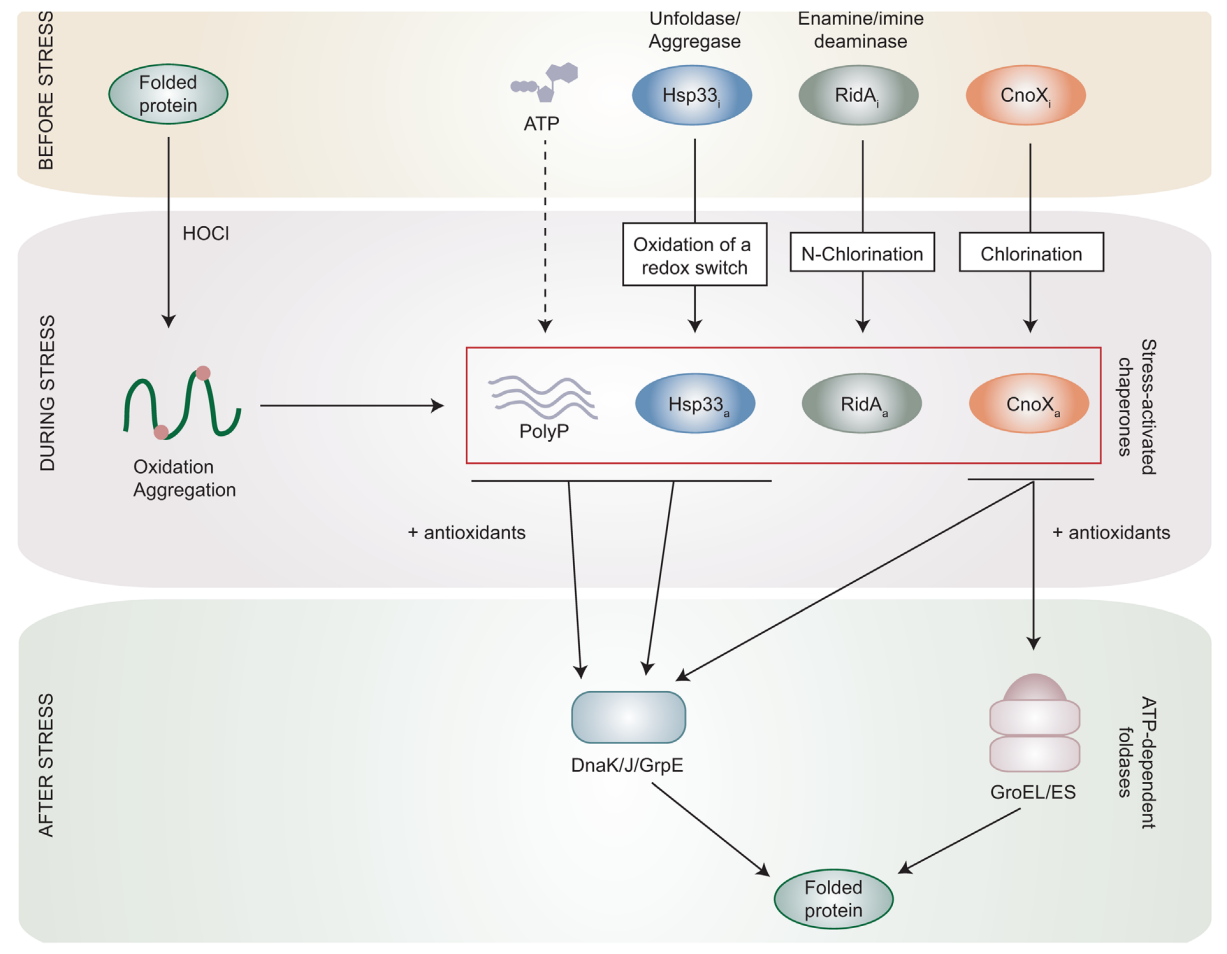
Protein protection network during hypochlorous acid (HOCl) stress. Upon HOCl stress, most proteins become oxidized and lose their three-dimensional structure, ultimately leading to their aggregation. In parallel, the oxidation or chlorination of stress-induced holdases (Hsp33, RidA, and CnoX) activates them upon HOCl stress, which allows them to bind and protect their substrates. Polyphosphate (PolyP), a chemical chaperone synthesized from ATP, has also been shown to bind unfolded proteins during stress. After stress, when the ATP pool is replenished and oxidative stress relieved, these stress-induced holdases cooperate with antioxidants to transfer their substrates to either DnaK/J/GrpE or GroEL/ES for proper refolding.

In the last decade, important insights into the mechanisms used by bacteria to mount effective, often complex responses against HOCl have been obtained. For instance, transcription factors that specifically respond to HOCl have been described in *E. coli* and other bacteria ^27^. They include HypT, which is activated through methionine oxidation ^28, 29^, and NemR, which is activated via cysteine oxidation. Furthermore, three HOCl-activated chaperones have been identified and shown to be important during HOCl stress. These chaperones are ATP-independent holdases, i.e. chaperones that prevent protein aggregation by binding unfolded proteins but do not promote protein refolding, and thus function during HOCl stress, when the ATP-dependent foldases, i.e. chaperones actively promoting protein refolding, are inactive ( Figure 2). In the following sections, we will briefly describe HOCl-activated chaperones and explain how they are activated under conditions that inactivate most other proteins ^30^.

### Hsp33

The first HOCl-activated chaperone identified was Hsp33, a protein which was recently described to work, under normal conditions, as an unfoldase/aggregase transferring EF-Tu to the Lon protease for degradation ^31^. However, when exposed to HOCl, Hsp33 is quickly transformed into a holdase through the oxidation of a redox switch involving four conserved, zinc-binding cysteine residues ^32– 38^. Oxidation of this redox switch induces structural changes in Hsp33 that now exposes hydrophobic surfaces and can interact with unfolded proteins ^32– 38^. Upon the cell’s return to normal conditions, oxidoreductases reduce Hsp33’s redox switch before its substrates are shifted to the ATP-dependent foldase DnaK/J/GrpE for refolding ^39, 40^ ( Figure 2).

### RidA

Another HOCl-activated chaperone is the *E. coli* protein RidA, for which the chaperone activity has been mostly studied *in vitro* ^41^. Interestingly, RidA, which normally functions as an enamine/imine deaminase involved in the synthesis of branched-chain amino acids ^42^, loses its deaminase activity when incubated with HOCl while it turns into a holdase via the reversible N-chlorination of positively charged residues, an unprecedented post-translational modification. N-chlorination makes the surface of RidA more hydrophobic, which activates its holdase activity ^41^ ( Figure 2). The fact that *ridA* mutant cells are more sensitive to HOCl ^41^ suggests that RidA protects *E. coli* against HOCl-induced damage. However, further investigation is required to determine the functional relevance of the HOCl-induced chaperone activity of this protein *in vivo* and its potential role in the proteostasis network under HOCl stress.

### CnoX

We recently identified CnoX as a novel type of protein folding factor that is essential for cell survival when *E. coli* is exposed to HOCl ^43^. We demonstrated that HOCl turns CnoX into a powerful holdase by chlorination in a mechanism similar to that described for RidA ^41^. Remarkably, CnoX can both function as a holdase and form mixed-disulfide complexes with client proteins. Under the latter role, CnoX prevents sensitive cysteine residues in its substrates from being irreversibly oxidized, which could otherwise have a detrimental effect on refolding and/or block reactivation. Because CnoX can solve two problems faced by proteins (aggregation and overoxidation), it has become the first member of a new class of proteins: the chaperedoxins ^43^. Importantly, we established that, after stress, CnoX is capable of transferring its substrates not only to DnaK/J/GrpE, like Hsp33 ^39^, but also to GroEL/ES, the only chaperone system essential for *E. coli* growth and survival ^44^. This feature is conserved in the *Caulobacter crescentus* CnoX homologue ^45^ ( Figure 2). CnoX is, to our knowledge, the first holdase shown to cooperate with GroEL/ES for protein refolding.

In addition to the proteins described above, work from the Jakob laboratory has led to the identification of PolyP, an inorganic polymer synthesized from ATP, as a chemical chaperone able to stabilize proteins during HOCl stress ^46^ ( Figure 2). Accordingly, intracellular levels of PolyP increase during HOCl stress, as a result of both decreased hydrolysis ^46^ and probably also increased synthesis, although this remains to be firmly established.

## Conclusions

Whereas the important role for reducing enzymes, such as catalases, peroxiredoxins, thioredoxins, and glutaredoxins, in fighting oxidative stress in bacteria has been known for some time, the crucial function of HOCl-induced chaperones for proteostasis has emerged more recently. The identification of an increasing number of these chaperones, in both prokaryotes and eukaryotes, raises a number of questions and hypotheses that will have to be addressed in the future. First, because activation by chlorination appears to be rather unspecific compared to activation by oxidation of cysteine residues, like in Hsp33, it is likely that additional proteins share the ability to be activated by HOCl. Supporting this, it was recently reported that a number of proteins from human blood plasma are converted into holdases by HOCl via N-chlorination ^47^. Second, the identified stress-induced chaperones are expressed under non-stress conditions and are conserved in a large number of organisms, including non-pathogenic bacteria that are less likely to be exposed to high levels of HOCl in their natural environment. It is therefore tempting to speculate that these proteins display a basal function under normal conditions but evolved in certain organisms to act as chaperones under specific stress conditions. Focusing on the CnoX chaperedoxin expressed by the aquatic bacterium *C. crescentus*, we recently found that, in contrast to its *E. coli* counterpart, it functions as a thioredoxin and a constitutive holdase that does not need to be activated by HOCl. Thus, within the family of CnoX proteins, only certain proteins (such as *E. coli* CnoX) have evolved to provide specific protection against HOCl stress ^45^. In the same line, it was recently shown that N-chlorination does not activate the homolog of RidA from *Staphylococcus aureus* into a chaperone ^48^. Thus, future work should determine the extent of the stress-induced chaperone network upon HOCl stress as well as the roles for these proteins under non-stress conditions and/or in non-pathogenic organisms.

## Abbreviations

H _2_O _2_, hydrogen peroxide; HOCl, hypochlorous acid; O _2_^•–^, superoxide radical; O _2_, molecular oxygen; ^•^OH, hydroxyl radical; MPO, myeloperoxidase; PolyP, polyphosphate; ROS, reactive oxygen species.
